# Is There an Association Between Vitamin D Deficiency and Erectile Dysfunction? A Systematic Review and Meta-Analysis

**DOI:** 10.3390/nu12051411

**Published:** 2020-05-14

**Authors:** Andrea Crafa, Rossella Cannarella, Rosita A. Condorelli, Sandro La Vignera, Aldo E. Calogero

**Affiliations:** Department of Clinical and Experimental Medicine, University of Catania, via S. Sofia 78, 95123 Catania, Italy; crafa.andrea@outlook.it (A.C.); rossella.cannarella@phd.unict.it (R.C.); sandrolavignera@unict.it (S.L.V.); acaloger@unict.it (A.E.C.)

**Keywords:** Vitamin D, erectile dysfunction, arteriogenic erectile dysfunction, hypogonadism, male sexual function

## Abstract

Erectile dysfunction (ED) is found very frequently in the male population, in particular in its arteriogenic form, which also represents an important predictor of cardiovascular diseases (CVDs). Some evidence suggests that vitamin D could play a role in cardiovascular risk prevention thanks to its ability to reduce endothelial damage, oxidative stress, the production of inflammatory cytokines, and dyslipidemia. Since ED and CVDs have pathogenic mechanisms in common, numerous studies have evaluated a possible association between vitamin D deficiency (blood concentrations of 25-hydroxyvitamin D < 20 ng/mL) and ED, but with conflicting results. This meta-analysis was therefore performed to clarify the discrepancy of the data so far published. To achieve this, articles have been searched extensively in the Pubmed, MEDLINE, Cochrane, Academic One Files, Google Scholar, and Scopus databases from the first day they were created until January 2020. The search strategy included pertinent Medical Subjects Headings (MeSH) terms. Of the 431 items retrieved, only eight observational studies were included, resulting in a total sample size of 4055 patients. It was found that 25-hydroxyvitaminD (25(OH)D) levels did not show any significant difference between patients with and without ED. However, when patients with vitamin D deficiency only were taken into account, the international index of erectile function (IIEF) score for erectile dysfunction was significantly worse than in controls. This association remained significant even when eugonadal-only patients were considered. Finally, we found that eugonadal patients with severe ED have lower 25(OH)D_3_ levels than patients with mild ED. In conclusion, this meta-analysis suggests an association between vitamin D deficiency and the presence of severe forms of ED, independent of testicular function.

## 1. Introduction

Vitamin D is a steroid hormone produced in the skin by 7-dehydrocholesterol, a cholesterol precursor, by the action of sunlight. It can also be introduced through the diet in the form of ergocalciferol (vitamin D_2_), present in organisms (mainly fungi), containing ergosterol, its precursor; or cholecalciferol (vitamin D_3_), of animal origin, which is the same product synthesized in the skin [1].

All these forms of vitamin D are inactive. Therefore, to carry out its action, vitamin D must be activated by going through a double hydroxylation. The first occurs in the liver in position C25, leading to the formation of 25-hydroxy-Vitamin D_3_ [25(OH)D_3_], the biological marker of circulating levels of vitamin D, also known as calcidiol or calcifediol. The second occurs in the kidney in position 1 [2]. Once activated, 1,25-dihydroxy-Vitamin D_3_ [1,25(OH)_2_ D_3_], also known as calcitriol, interacts with its nuclear receptor that is widely distributed in cells throughout the body. Due to the ubiquitous expression of the vitamin D receptor (VDR), the idea that vitamin D not only acts on the calcium-phosphorus metabolism but also has a number of non-calcemic effects, is growing [3]. For this reason, an increasing number of studies are being conducted to evaluate the effects of vitamin D deficit, even taking into account the high prevalence of this problem. Indeed, it has been estimated that 20–100% of U.S., Canadian and European elderly men and women have low levels of 25(OH)D_3_. The Endocrine Society defines vitamin D deficiency when the blood levels of 25(OH)D_3_ are lower than 20 ng/mL and vitamin D insufficiency for levels ranging from 21 to 29 ng/mL [4].

Recent findings have assigned to vitamin D a role in andrology. Accordingly, the microsomal enzyme cytochrome P450, subfamily 2R, polypeptide 1 (CYP2R1), responsible for vitamin D hydroxylation in position 25, is expressed not only in the liver, but also at the testicular level. In particular, the testicular tissue of healthy men has a five-fold higher expression of CYP2R1 mRNA compared with the liver. Specifically, this enzyme is expressed in Leydig cells in a luteinizing hormone (LH)-dependent manner [5]. Indeed, human chorionic gonadotropin (hCG) administration to male patients with central hypogonadism is also able to restore blood 25(OH)D_3_ levels, whereas testosterone replacement therapy is not. This confirms that the hydroxylation of vitamin D within the Leydig cells occurs with an LH-dependent mechanism. For these reasons, low blood concentrations of 25(OH)D_3_ may be associated with male hypogonadism [6]. 

With the exception of hypogonadism, it is not yet known whether vitamin D deficiency is associated with other andrological diseases. Recent data have been published to evaluate the role of vitamin D deficiency on erectile dysfunction (ED) [7], but the evidence is still contradictory. This is probably due to the heterogeneity of the studies published so far on this topic. Indeed, almost all of these studies included a cohort of patients with important comorbidities (e.g., diabetes mellitus, renal failure, hypogonadism, benign prostatic hyperplasia) [8,9,10,11,12,13,14,15,16,17,18], which in themselves are well-known causes of ED. Furthermore, very few studies have evaluated the effects of vitamin D supplementation on sexual dysfunction [13,14,15,16]. Therefore, the purpose of this meta-analysis was to evaluate the levels of 25(OH)D_3_ among patients with and without ED and if the severity of ED is different in patients with and without vitamin D deficiency. Possible sources of biases were considered with particular attention. The data were also analyzed excluding patients with hypogonadism, one of the main causes of ED.

## 2. Materials and Methods

### 2.1. Source

This study was performed by applying the MOOSE (Meta-analysis of Observational Studies in Epidemiology) Guidelines for Meta-analyses and systematic reviews of observational studies [19]. All data were extracted through extensive research in Pubmed, MEDLINE, Cochrane, Academic One Files, Google Scholar, and Scopus databases from their inception to January 2020. 

The search strategy included the following combination of Medical Subjects Headings (MeSH) terms and keywords: “vitamin d”, “25-hydroxy-Vitamin D”, “25 hydroxycholecalciferol”, “cholecalciferol”, “erectile dysfunction” (or “function”), “impotence”, “hypogonadism”, “sexual function” (or “dysfunction”). Other articles were selected manually using the reference lists of the articles found by entering the aforementioned keywords. In case of doubts regarding the data of these articles, we contacted the authors for clarification.

### 2.2. Study Selection

All the studies evaluating the association between vitamin D and ED by comparing either 25(OH)D_3_ levels in patients with ED and in controls or by evaluating the difference in the International Index of Erectile Function (IIEF) scores in patients with or without vitamin D deficiency were included in the quantitative synthesis. Some studies presented both data.

We excluded from the analysis those studies that did not allow the extraction of these data. We did not also include the articles reporting a therapeutic intervention aimed at improving the levels of 25(OH)_3_ (vitamin D supplementation), hormonal levels (testosterone replacement therapy) or erectile function (PDE5 inhibitors). The study-design included: observational studies, whereas reviews, comments, case-reports and animal studies were excluded. 

After carrying out the first analysis of all the studies, we performed a further analysis excluding all the articles including patients with hypogonadism. In this way, we assessed whether low levels of 25(OH)D_3_ were only an epiphenomenon of testicular dysfunction which has a profound impact on ED. 

The quality assessment of the studies included in the present meta-analysis was independently performed by two authors (A.C. and R.C) using the Newcastle-Ottawa scale (NOS), which evaluates the following three distinct domains: (a) selection; (b) comparability; (c) exposure or outcome. The maximum score is 9. Studies with a score of <5 were considered to have a high risk of bias, between 5 and 7 moderate risk, >7 low risk of bias [20]. Any disagreement between the two investigators was resolved through discussion with a third investigator (A.E.C).

For each endpoint examined, the mean difference (MD) or the standardized mean difference (SMD) and the 95% confidence intervals (CI) were calculated. SMD was used for the analysis of the IIEF parameter since not all studies used the IIEF-5 to evaluate the severity of ED. In fact, some authors have used the IIEF-15, reporting only the sum of the 6 items that explore the erectile function domain. The formula suggested by Hozo and colleagues [21], which allows estimating the mean and standard deviation (SD) having only the median and range values, was used for the studies whose data are reported as median and range and it was not possible to have those values from the authors.

The Cochran-Q and I^2^ statistics were used to evaluate the statistical heterogeneity. Specifically, if I^2^ was lower or equal to 50%, the variation of the studies was considered to be homogenous and the fixed effect model was adopted. If I^2^ was higher than 50%, there was significant heterogeneity between studies and the random-effects model was used. All *p*-values lower than 0.05 were considered statistically significant. The analysis was performed using RevMan software v. 5.3 (Cochrane Collaboration, Oxford, UK).

## 3. Results

**Figure 1:**
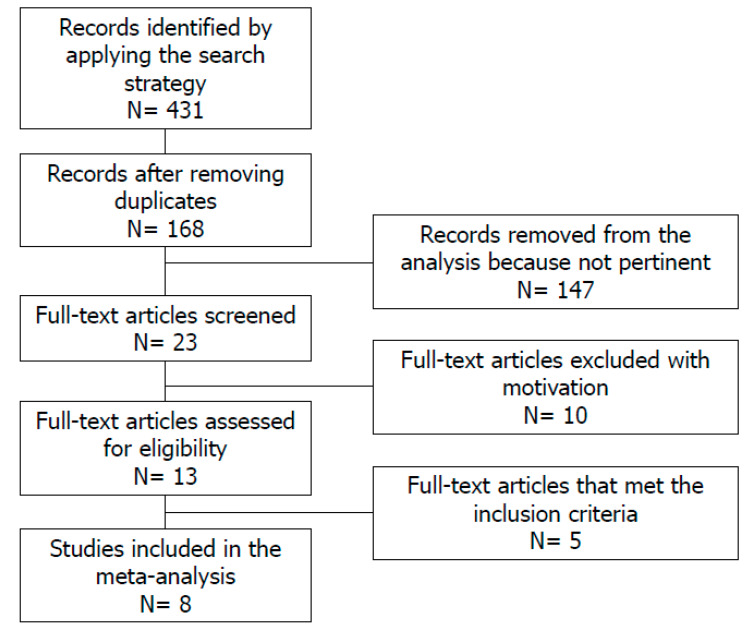
Flowchart of the studies included in the meta-analysis.

The aforementioned search strategy identified a total of 431 records. After the exclusion of duplicates, the remaining 168 articles were screened. Of these, 147 were judged not pertinent after reading their titles and abstracts. The residual 23 full texts were carefully read. Of these, 10 were excluded because they were reviews (n = 4), comments (n = 4), or animal studies (n = 1). Therefore, 13 were found suitable for inclusion in this study. Three of these latter studies were excluded for the study design: they were intervention studies [13,14,15]. The articles by Bellastella and colleagues [17] and Zhang and colleagues [18] were excluded because we could not extract useful data for the meta-analysis. We also contacted the latter authors for clarification, but received no response. Finally, eight articles [8,9,10,11,12,22,23,24] met our inclusion criteria and were therefore included in the analysis, resulting in a total sample size of 4055 patients (Figure 1). The main characteristics of the studies included in the meta-analysis are summarized in Table 1. Furthermore, all the articles scored between 5–7 on the NOS, thus they classified as studies with moderate risk of bias (Table 2).

**Figure 2:**
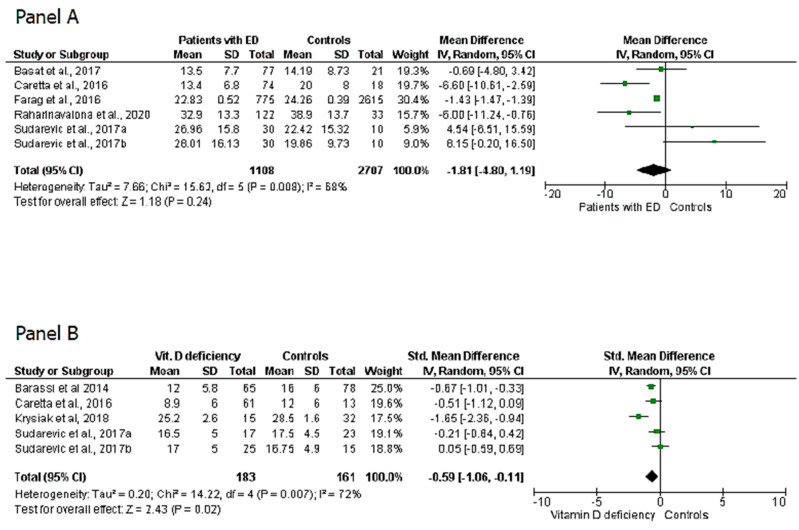
(**Panel A**) 25-hydroxy-Vitamin D levels in patients with erectile dysfunction and controls. (**Panel B**) International index of erectile function-5 scores in patients with Vitamin D deficiency vs. patients with vitamin D insufficiency or sufficiency.

**Figure 3:**
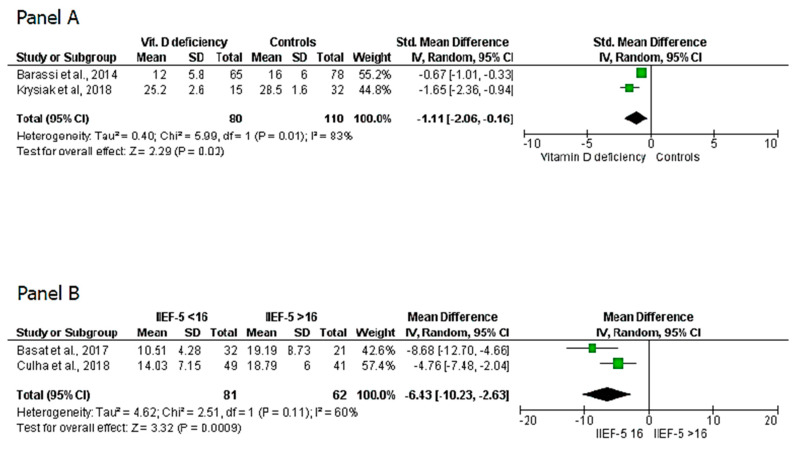
(**Panel A**) International index of erectile function-5 (IIEF-5) scores in patients with 25-hydroxy-Vitamin D <20 ng/mL vs. patients with 25-hydroxy-Vitamin D ≥20 ng/mL. (**Panel B**) 25-hydroxy-Vitamin D levels in patients with IIEF-5 score <17 vs. patients with 17 <IIEF-5 score ≤25.

The eight articles selected for this study were then classified into four sets: (a) articles evaluating 25(OH)D_3_ levels in patients with or without ED [8,9,10,11,12]; (b) articles evaluating the IIEF-5 score in patients with or without vitamin D deficiency [8,12,22,23]; (c) articles that excluded patients with hypogonadism and evaluated the IIEF-5 score in patients with or without vitamin D deficiency [22,23]; (d) articles that excluded patients with hypogonadism and assessed whether patients with ED and worse IIEF-5 scores (<16) had lower 25(OH)D_3_ levels than patients with ED and higher IIEF-5 score (>16) [10,24]. Some studies fell into more than one category. Meta-analysis was performed separately for each category of data generated by this classification. Sudarevic et al.’s study has been included twice in the analysis since the measurements taken in summer and winter have been independently considered [19].The analysis of the five studies (six considering the two measurements of Sudarevic et al., 2017) of the first set showed that patients with ED do not have significantly different levels of 25(OH)D_3_ than controls (MD = 1.81 ng/mL; 95% CI −4.8, −1.19; P = 0.24) (Figure 2, panel A). However, the analysis of the four studies (five considering Sudarevic et al., 2017) of the second set showed that the mean IIEF-5 score is significantly lower in patients with vitamin D deficiency than in those with insufficient or adequate levels (SMD = 0.59; 95% CI −1.06, −0.11; P = 0.02) (Figure 2, panel B). The statistical significance was confirmed when only the studies that excluded patients with hypogonadism (third set) were considered (SMD = 1.11; 95% CI −2.06, −0.16; P = 0.01) (Figure 3, panel A). Finally, the analysis of the fourth set of data showed that eugonadal patients with ED and worse IIEF-5 scores had very lower levels of 25(OH)D_3_ than eugonadal patients with ED and higher IIEF-5 scores (SMD = −6.43; 95% CI −10.23, −2.63; P = 0.0009) (Figure 3, panel B).

## 4. Discussion

ED is a widespread problem resulting from psychological and/or organic causes. Among the latter, undoubtedly, the etiology with the highest prevalence is the vascular form [25], which is also a predictive factor for cardiovascular disease (CVD). Indeed, arteriogenic ED and CVD share common risk factors that include aging, blood hypertension, diabetes mellitus, insulin-resistance, cigarette smoking, increased body mass index (BMI), hypercholesterolemia, and lower high-density lipoprotein (HDL) [26]. Several studies have evaluated the association between vitamin D and cardiovascular risk and the effects of vitamin D supplementation on this risk, often with contradictory results. A recent meta-analysis including 81 randomized clinical trials selected through stringent criteria, highlighted the ability of vitamin D supplementation to improve some cardiovascular risk factors including elevated blood pressure, hyperparathyroidism, dyslipidemia and inflammation [27]. Considering the association between ED and CVD and the supposed positive effects of vitamin D supplementation on cardiovascular risk factors, some authors have tried to understand if there is also an association between vitamin D and ED. However, even in this case the results have been contradictory. 

Several mechanisms have been explored to explain the association between vitamin D deficiency and ED. Undoubtedly the main link between the two conditions appears to be the vascular endothelium. A dysfunctional endothelium has a reduced vasodilatory ability associated with an increase in the pro-inflammatory and pro-thrombotic state [28]. Vitamin D plays a role in endothelial integrity improving the function of endothelial cells by promoting repair mechanisms [29]. Moreover, it protects endothelial cells, promoting an anti-inflammatory state and reducing oxidative stress. Indeed, vitamin D seems to have antioxidant effects by reducing the expression of NADPH oxidase and promoting the action of other antioxidant enzymes and molecules such as superoxide dismutase, glutathione peroxidase, catalase, ascorbic acid, α-tocopherol, and glutathione that act by counteracting free radicals [30]. In addition, a study in diabetic rats showed the ability of maxacalcitol, an analogue of vitamin D, to improve the antioxidant pathway of nuclear factor erythroid 2-related factor 2 (Nrf2) [31]. Inflammation can exert its detrimental action on the endothelium through both chronic low-grade inflammation, associated with atherosclerotic mechanisms, and by the acute systemic inflammatory response [32]. Vitamin D exerts an immunomodulatory action thus reducing inflammation. It inhibits the pro-inflammatory activity of CD4+ Th1 cells resulting in a reduction of inflammatory cytokines such as IL-2, interferon (IFN)-γ, and tumor-necrosis factor-α. Moreover, vitamin D also promotes Th2 responses by enhancing IL-4, IL-5, and IL-10 production. Finally, it could increase regulatory T cell activity and suppress Th17 responses. On this basis, vitamin D promotes a transition from an inflammatory Th1 response to an anti-inflammatory Th2 response [33]. In more detail, vitamin D seems to have an inhibitory effect on the nuclear factor kB (Nf-kB) pathway involved in the expression of several pro-inflammatory mediators such as TNFα, IL-1, and IL-6. In addition, vitamin D exerts a direct inhibitory effect on the expression of TNFα receptors 2 and 4, thus preventing the inhibitory action of this mediator on endothelial nitric oxide synthetase (eNOS) and its ability to promote inflammation through the further activation of the NF-kB [30].

Another mechanism associating vitamin D deficiency with ED is the mean platelet volume (MPV). This is the most commonly used measure of platelet size and is a marker of their reactivity. Indeed, larger platelets are metabolically and enzymatically more active with a greater prothrombotic potential leading to increased platelet aggregation, thromboxane synthesis, and increased expression of adhesion molecules. All these factors increase the risk of vascular diseases including CVD and ED [34]. The evidence suggests that patients with vitamin D deficiency have a higher MPV and therefore a greater risk of vascular disease. Indeed, Parker and colleagues showed that vitamin D deficient patients had significantly higher MPV than those with adequate 25(OH)D_3_ D3 levels [35]. Similarly, Korzonek-Szlacheta and colleagues found that the MPV in patients with 25(OH)D_3_ levels >20 ng/mL was lower than that in patients with 25(OH)D_3_ levels <10 ng/mL and in patients with 25(OH)D_3_ levels comprised between 10 and 20 ng/mL [36]. 

Finally, it has been shown that vitamin D is able to stimulate the expression eNOS and therefore the production of nitric oxide (NO), a key molecule in the erectile response. Hence the reduction of NO production in patients with vitamin D deficiency could be another mechanism that explains its correlation with ED [32].

The present meta-analysis apparently confirms the conflicting evidence of literature on the role of vitamin D in ED. 25(OH)D_3_ levels in patients with and without ED did not show any statistically significant difference between the two groups. However, patients with vitamin D deficiency had lower IIEF scores, hence a greater ED severity, than patients with adequate levels of this hormone. However, the heterogeneity and limitations of the studies included in the analysis must be taken into due consideration before drawing any conclusion. In fact, no studies reported whether patients had undergone echo-color Doppler to diagnose the presence of an arteriogenic ED, except for the study by Barassi and colleagues [23]. These authors showed that patients with arteriogenic ED had 25(OH)D_3_ levels significantly lower than patients with other forms of ED [23]. This would seem consistent with the evidence suggesting a role for vitamin D deficiency in inducing endothelial damage, which is one of the main pathophysiological mechanisms of arteriogenic ED [37]. Furthermore, vitamin D deficiency appears to be associated with a dyslipidemic profile, which is another risk factor for arteriogenic ED [38]. Therefore, the heterogeneity of the cohorts of patients with ED included in the studies attempting to establish a relationship with 25(OH)D_3_ levels could explain the lack of statistical significance that we found when all the studies were included in the meta-analysis (Figure 2, panel A). In contrast, the statistical significance found in the second series of studies (Figure 2, panel B), when only patients with vitamin D deficiency are considered, agrees with the alleged association between vitamin D deficiency and vascular ED. In fact, our data seem to confirm the evidence suggesting that lower IIEF scores are more often associated with organic forms and, as already mentioned, the arteriogenic form is the most frequent [39]. Another important aspect to mention is that the patients studied are of different ages and it is well known that the prevalence of both ED [26] and vitamin D deficiency [4] increases with age. This represents another important bias for the correct interpretation of the data. Furthermore, it is noteworthy that the serum levels of 25(OH)D_3_ have a seasonal variability [40] and only one study has clarified whether 25(OH)D_3_ was measured in summer or winter. Therefore, the bias of seasonality could be excluded [8].

Furthermore, the populations examined in the studies used for the meta-analysis are often patients with comorbidities, which are confounding factors for the correct interpretation of the association between vitamin D and ED. Indeed, half of the studies were conducted on patients with diabetes mellitus [9,10,11,12]. In this regard, a recent meta-analysis including 145 studies reported an ED prevalence of 59% in diabetic patients who have an odds ratio of 3.62 to develop ED compared to controls. Different mechanisms are involved in the pathogenesis of diabetes-related ED, including neuropathy, micro- and macrovascular arterial disease, hypogonadism, psychogenic components and drug side effects [41]. For this reason, the exact burden of vitamin D deficiency in the pathogenesis of ED is difficult to estimate in patients with diabetes. However, some studies have suggested a role of vitamin D deficiency in the development of diabetes and its complications. In fact, VDR is expressed in fat and muscle cells where it would improve insulin sensitivity and insulin-resistance. It is also expressed in β-cells where it would contribute to their proper function. Furthermore, thanks to its anti-inflammatory and antioxidant properties, vitamin D seems to lower the prevalence of micro and macro-vascular complications and neuropathy [42]. On this basis, vitamin D could play a role in preventing diabetes and therefore ED, which in a proportion of cases is the first sign of undiagnosed diabetes [41].

Sudarevic and colleagues conducted a study on patients who underwent kidney transplantation following the development of chronic kidney disease (CKD). In this cohort of patients, they did not find any association between vitamin D and ED [8]. Some evidence suggests that the prevalence of ED in CKD patients is around 50–80% and this prevalence remains high even after transplantation, albeit with a slight improvement. As for diabetes, the genesis of ED in CKD patients is multifactorial. Indeed, hormonal abnormalities (decrease of free and total testosterone levels, hyperprolactinemia, decline of erythropoietin levels), neuropathy associated with uremia, endothelial dysfunction, psychological factors and drugs (first of all diuretics) occur in these patients [43]. Furthermore, other factors playing a role in the pathogenesis of ED might be present in transplanted patients, such as a decrease of the cavernous blood flow following the end-to-end arterial anastomosis with the internal iliac artery [44] or the end-to-side external iliac artery anastomosis [45]. Considering this multi-factoriality, vitamin D deficiency can only have a minor influence on ED in these patients. In addition, 17.5% of the patients included in the study by Sudarevic and colleagues had already been treated with vitamin D after enrollment, thus influencing the final data and, accordingly, any association with ED [8].

Another relevant aspect that has introduced a strong bias in evaluating the relationship between vitamin D deficiency and ED is the presence of patients with hypogonadism in the cohort studied. Indeed, only a few studies have excluded hypogonadism [10,22,23,24]. In these patients, low testosterone levels are associated with ED, hypoactive sexual desire and low frequency of nocturnal erections. A study identified testosterone threshold values for the onset of each symptom. In detail hypoactive sexual desire appears for values below 8 nmol per liter (2.3 ng per milliliter), ED shows up for values below 8.5 nmol (2.5 ng per milliliter), finally decreased frequency of morning erections occurs for values lower than 11 nmol per liter (3.2 ng per milliliter) [46]. A recent meta-analysis showed that testosterone replacement therapy improves erectile function and is often able to resolve milder forms of ED, while additional treatments are needed for more severe forms [47]. Late-onset hypogonadism (LOH) is a condition associated with age. Indeed, testosterone levels decrease by 0.4–2% per year starting from the third decade [48]. This decrease is accelerated in the presence of higher BMI and greater waist circumference, as well as in the presence of chronic diseases such as diabetes and kidney failure [46,47,48,49]. As previously mentioned, hypogonadism is also associated with low levels of 25(OH)D_3_ [5]. To make sure that the studies showing an association between vitamin D and ED were not conditioned by the presence of patients with hypogonadism, we performed a sub-analysis including only the studies that had hypogonadism as an exclusion criterion. Although only a few studies have taken this aspect into consideration, the sub-analysis showed that vitamin D is associated with ED regardless of testosterone levels. This same result was found in the sub-analysis of the study by Farag and colleagues [11] conducted on 562 men with normal testosterone levels. They showed that the association between 25(OH)D_3_ levels and ED found in the initial analysis they performed, was also stronger when only eugonadal patients were considered. In the last years, although with conflicting results, there is evidence that vitamin D supplementation could increase serum total testosterone or bioavailable testosterone levels by modulating the serum levels of sex hormone-binding globulin levels (SHBG). Therefore, this effect on testosteronemia could be another mechanism by which vitamin D might improve sexual performance [50].

Mostafa and colleagues also suggested a possible role of vitamin D deficiency in predicting the response to phosphodiesterase 5 inhibitors (PDE5i). They found that significantly lower levels of vitamin D are found in patients with ED non-responders to sildenafil. However, the study presents some important biases, including the absence of an evaluation of serum testosterone levels and the lack of diagnosis between the various forms of ED [51]. Furthermore, no interventional studies have been published on the response to PDE5i after vitamin D supplementation. Certainly, this type of study is necessary to better understand the role of vitamin D in the PDE5i responsiveness.

Indeed, more interventional studies are needed to better understand the role of vitamin D on ED. The few studies of this type have given conflicting results. In fact, Tirabassi and colleagues have shown that vitamin D supplementation, given until normal circulating levels were restored, is associated with an improvement in ED that is independent of circulating testosterone levels [13]. Similarly, Canguven and colleagues estimated that supplementation with 600,000 units of ergocalciferol per month for 12 months is associated with a progressive improvement in ED and an increase of serum testosterone levels [14]. In contrast, Kydir and colleagues reported that although low 25(OH)D_3_ levels are associated with poorer sexual performance in dialysis patients, there was no significant improvement of the sexual dysfunction after vitamin D administration [15]. Similarly, sexual function did not improve in a previous study in patients with renal insufficiency undergoing dialysis [16].

To date, this is the second meta-analysis on this topic. Moreover, although only one more study was included compared to the previously published meta-analysis [7], we found partly different results, probably due to the difference in data extraction from the studies selected [8,12,18]. For the first time, we report an analysis that excludes patients with hypogonadism whose inclusion represents, from our point of view, a major bias. 

In conclusion, although the data must be interpreted with great caution, due to the low quality and the small number of studies that could be selected, this meta-analysis suggests a role for vitamin D in conditioning the severity of ED that also seems to be independent of serum testosterone levels. Particularly, this study suggests an association between vitamin D deficiency with only the most severe forms of ED which, as above-mentioned, are more likely associated with an organic etiopathogenesis and therefore mainly arteriogenic forms [32]. This is also confirmed by our analysis of the fourth set of studies (Figure 3, panel B) which showed extremely lower 25(OH)D_3_ levels in eugonadal patients and with worse IIEF-5 score. However, multiple randomized clinical trials evaluating the effects of vitamin D supplementation in eugonadal patients would be helpful in demonstrating this hypothesis, particularly if they will be focused on arterial ED.

## Figures and Tables

**Table 1:** Summary of the studies included.

Source	Sample Size	No. of ED Patients	No. of Control	25(OH)D_3_ Levels in ED Patients	25(OH)D_3_ Levels in Control Group	No. of Vitamin Ddeficiency Patients	No. of Vitamin D Normal Patients	IIEF Score in Vitamin D Deficiency Patients	IIEF Score in Patients with Normal Levels of 25(OH)D_3_ or with Insufficiency
Farag et al., 2016	3390	775	2615	22.83 ± 0.52	24.26 ± 0.39	/	/	/	/
Basat et al., 2017	98	77	21	13.54 ± 7.24	14.19 ± 8.73	/	/	/	/
Culha et al., 2018	90	90	/	18.79 ± 6 in mild ED 14.03 ± 7.15 in severe ED	/	/	/	/	/
Krysiak et al., 2018	47	11	36	/	/	15	32	25.2 ± 2.6	28.5 ± 1.6
Barassi et al., 2014	143	143	/	18.2 ± 8.07 (A-ED) 22.5 ± 5.3 (BL-ED) 25.3 ± 11.6 (NA-ED)	/	65	78	12 ± 5.8	16 ± 4
Sudarevic et al., 2017	40	30	10	26.96 ± 15.8 (summer value) 28.01 ± 16.13 (winter value)	22.42 ± 15.32 (summer value) 19.86 ± 9.73 (winter value)	17 Summer 25 Winter	23 Summer 15 winter	16.5 ± 5 (summer value) 17 ± 5 (winter value)	17.5 ± 4.5 (summer value) 16.75 ± 4.9 (winter value)
Raharinavalona et al., 2020	155	122	33	32.9 ± 13.3	38.9 ± 13.7	/	/	/	/
Caretta et al., 2016	92	74	18	13.4 ± 6.8	20 ± 8	34	13	8.9 ± 6	12 ± 6

**Table 2:** Evaluation of the quality of studies using the Newcastle-Ottawa scale NOS.

Source	Study Design	No. of ED Patients	No. of Controls	No. of Vitamin D Deficiency Patients	No. of Vitamin D Normal Patients	Outcomes	Selection	Comparability	Exposure or Outcome	Risk of Bias
Farag et al. 2016 (11)	Cross-sectional	775	2615	/	/	Difference in 25(OH)D_3_ levels in patients with and without ED	***	*	*	Moderate
Basat et al. 2017 (10)	Cross-sectional	77	21	/	/	Difference in 25(OH)D_3_ levels in patients with and without ED	***	*	*	Moderate
Krysiak et al. 2018 (22)	Cross-sectional	11	36	15	32	Difference in IEFF-15 score in patients with or without vitamin D deficiency	**	*	**	Moderate
Barassi et al. 2014 (23)	Cross-sectional	143	/	65	78	Difference in 25(OH)D_3_ levels in patients with different form of ED and difference in IIEF-5 score in patients with or without vitamin D deficiency	***	**	*	Moderate
Sudarevic et al., 2017 (8)	Cross-sectional	30	10	17	23	Difference in 25(OH)D_3_ levels in patients with and without ED and difference in IIEF-5 score in patients with or without vitamin D deficiency	**	*	**	Moderate
Raharinavalona et., 2020 (9)	Cross-sectional	122	33	/	/	Difference in 25(OH)D_3_ levels in patients with and without ED	***	*	**	Moderate
Culha et Al 2018 (24)	Cross-sectional	90	/	/	/	Difference in 25(OH)D_3_ levels in patients with severe form of ED than patients with mild form	***	*	**	Moderate
Caretta et al. 2016 (12)	Cross-sectional	74	18	34	13	Difference in 25(OH)D_3_ levels in patients with and without ED and difference in IIEF-5 score in patients with or without vitamin D deficiency	***	**	*	Moderate

The Newcastle-Ottawa scale (NOS), evaluates the following three distinct domains: (1) selection; (2) comparability; (3) exposure or outcome. Each domain can receive a maximum of 3 points, shown with asterisks, up to a maximal total score of 9 points.
